# Immune monitoring of neoadjuvant chemo-immunotherapy for triple-negative breast cancer

**DOI:** 10.3389/fimmu.2025.1654748

**Published:** 2025-10-15

**Authors:** Munnawara Fatima Syeda, Rita Antunes Santos, Luana Madalena Sousa, Ana Raquel Paiva, Noémia Castelo Branco, Tatiana Cunha Pereira, Gabriela Sousa, Paulo Rodrigues-Santos

**Affiliations:** ^1^ Laboratory of Immunology and Oncology, Centre for Neuroscience and Cell Biology (CNC), University of Coimbra, Coimbra, Portugal; ^2^ Faculty of Medicine (FMUC), University of Coimbra, Coimbra, Portugal; ^3^ Medical Oncology Department, Portuguese Institute of Oncology of Coimbra Francisco Gentil (IPOCFG EPE), Coimbra, Portugal; ^4^ Clinical Pathology Department, Portuguese Institute of Oncology of Coimbra Francisco Gentil (IPOCFG EPE), Coimbra, Portugal; ^5^ Anatomic Pathology Service, Portuguese Institute of Oncology of Coimbra Francisco Gentil (IPOCFG EPE), Coimbra, Portugal; ^6^ Institute of Immunology, Faculty of Medicine (FMUC), University of Coimbra, Coimbra, Portugal; ^7^ Research Centre in Environment, Genetics and Oncobiology (CIMAGO), Faculty of Medicine, University of Coimbra, Coimbra, Portugal; ^8^ Coimbra Institute for Biomedical Research (iCBR), Faculty of Medicine, University of Coimbra, Coimbra, Portugal; ^9^ Centre for Innovation in Biomedicine and Biotechnology (CIBB), University of Coimbra, Coimbra, Portugal; ^10^ Clinical Academic Center of Coimbra (CACC), Coimbra, Portugal

**Keywords:** triple-negative breast cancer, neoadjuvant therapy, chemotherapy, cancer immunotherapy, immune monitoring

## Abstract

Triple-negative breast cancer (TNBC) is an aggressive subtype defined by the absence of estrogen receptor, progesterone receptor, and HER2 expression. It accounts for 10–20% of breast cancer cases, predominantly affecting younger women, and is associated with poor prognosis due to high recurrence rates and limited therapeutic options. Past treatment strategies relied solely on chemotherapy, but challenges such as metastatic potential and chemoresistance persisted. Recent advancements in neoadjuvant chemo-immunotherapy aim to address these limitations by combining chemotherapy with immune checkpoint inhibitors, with promising clinical trial results demonstrating improved response rates and survival outcomes. A central focus is placed on biomarker-based immune monitoring strategies, encompassing both tissue-based biomarkers—such as programmed cell death ligand 1 (PD-L1) expression, microsatellite instability, tumor mutational burden, tumor-infiltrating lymphocytes (TILs), and gene expression signatures—and blood-based biomarkers, including gene expression profiling, comprehensive immunophenotyping, and cytokine profiling. In addition, an emerging role of advanced imaging technologies, such as immuno-positron emission tomography (immuno-PET) and radiomics, could permit real-time immune monitoring. This review aims to provide a comprehensive overview of the current landscape of immune monitoring in TNBC, highlighting its challenges, predictive and prognostic value, and potential to guide clinical decision-making. By addressing key immune response biomarkers, technical limitations, and emerging technologies, we seek to outline strategies for optimizing treatment and enhancing personalized medicine approaches for TNBC patients. Future integration of innovative monitoring techniques holds promise for improving patient outcomes.

## 1 Introduction

### 1.1 Definition and characteristics

**Figure 1:**
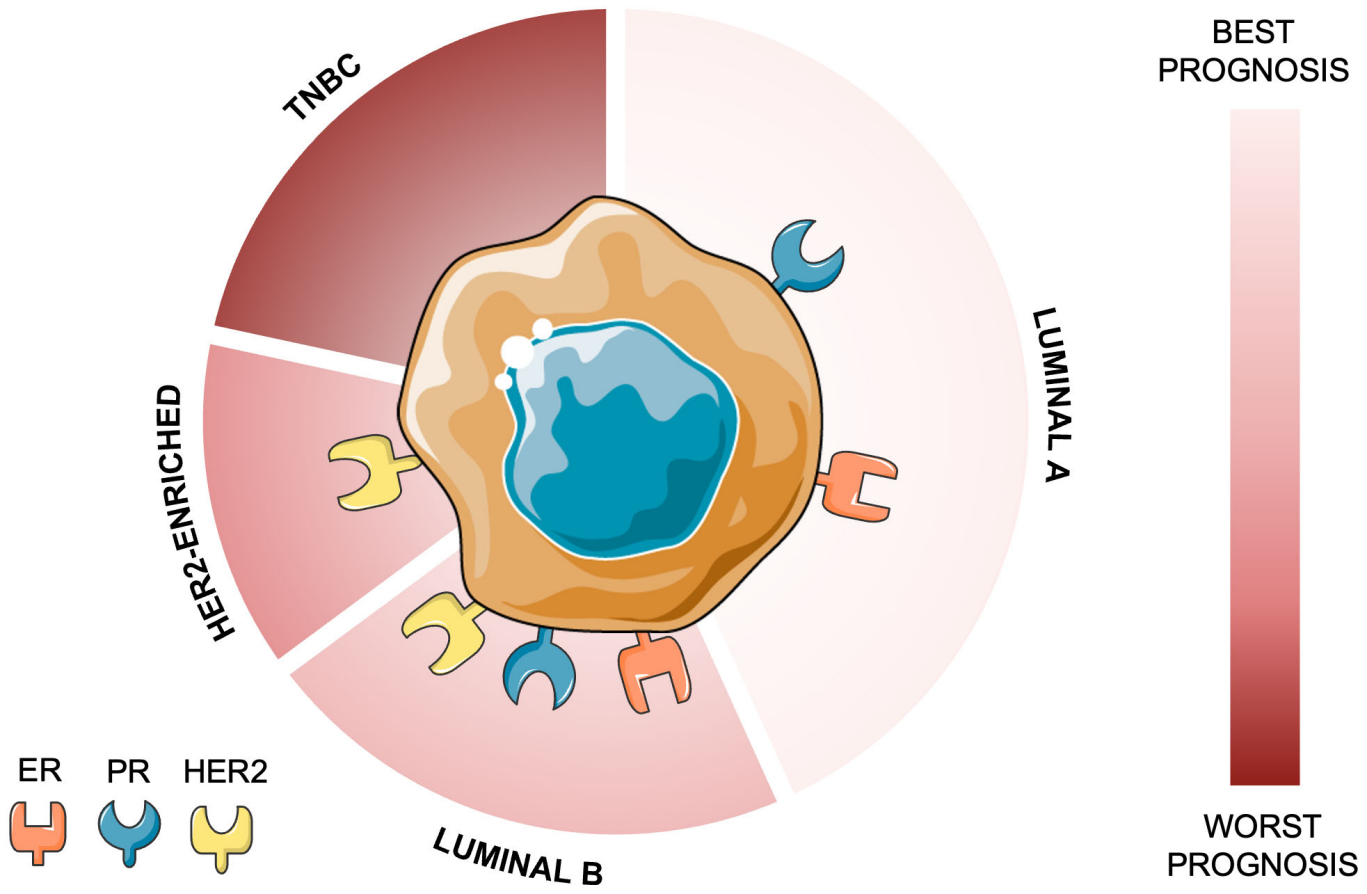
Classification of main molecular subtypes of breast cancer based on receptor status. Luminal A (ER+ and PR+, HER2-, low Ki-67), is hormone receptor-positive and typically responsive to endocrine therapy; Luminal B (ER+ and/or PR+, HER2+/- or high Ki-67), may also benefit from HER2-targeted therapies; HER2-Enriched (ER-/PR-, HER2+), is characterized by high HER2 expression and sensitivity to HER2-targeted therapies; and Triple-Negative Breast Cancer (TNBC) (ER-, PR-, HER2-), an aggressive subtype lacking hormone and HER2 receptors, often requiring chemotherapy as the primary treatment option. This classification is critical for determining appropriate therapeutic strategies. ER – estrogen receptor; HER2 – Human epidermal growth factor 2; PR – progesterone receptor.

Triple-negative breast cancer (TNBC) is defined by the lack of estrogen receptor (ER), progesterone receptor (PR), and human epidermal growth factor receptor 2 (HER2) expression. Certain special histological types (TNBC ST) show a more favorable prognosis. Low-grade TNBC STs include salivary gland-like tumors (classic adenoid-cystic, low-grade mucoepidermoid, secretory carcinomas), mucinous cystadenocarcinoma, tall cell carcinoma with reversed polarity, and low-grade metaplastic carcinomas. High-grade TNBC STs, including high-grade adenoid-cystic, polymorphous, and high-grade metaplastic carcinomas, are more aggressive. HER2-negative apocrine carcinomas have uncertain prognosis. Low-grade TNBC STs and LAR or BL1 subtypes often exhibit better clinical outcomes than classic TNBC (1). TNBC is a heterogeneous disease, not only histopathologically but also at the molecular level. Lehmann et al. (2) classified into four distinct subtypes with therapeutic implications. The basal-like 1 (BL1) subtype is enriched in DNA damage response pathways and shows high sensitivity to platinum chemotherapy and poly (ADP-ribose) polymerase (PARP) inhibitors. Basal-like 2 (BL2) displays growth factor signaling and glycolytic activity, with limited chemotherapy response. The mesenchymal (M) subtype is characterized by epithelial-mesenchymal transition (EMT) and stemness pathways, suggesting vulnerability to phosphatidylinositol 3-kinase/mammalian target of rapamycin (PI3K/mTOR) pathway inhibition. Finally, the luminal androgen receptor (LAR) subtype exhibits androgen-driven signaling, responsive to anti-androgen therapies. TNBC is characterized by its distinctive molecular profile, aggressiveness, different metastatic patterns, and absence of targeted therapy (**Figure 1**). TNBC accounts for ~10-20% of invasive breast cancers (BC), with an estimated 170,000 instances worldwide (3, 4).

In contrast to hormone receptor (HR)-positive or HER2-positive disease, TNBC has a significantly more aggressive clinical evolution, characterized by an earlier onset of symptoms, a larger propensity for metastasis, and poorer clinical outcomes, as seen by elevated rates of relapse and decreased survival rates (5, 6). According to histological findings, most TNBCs are of ductal origin; however, numerous other aggressive phenotypes appear to be over-represented, including metaplastic, apocrine, and adenoid cystic (7). A histological examination of basal-like tumors, all of which were ER/HER2 negative, revealed a significant rise in mitotic count, geographic necrosis, invasion borders, and stromal lymphocytic response (8).

### 1.2 Epidemiology

A particularly significant fact is that African American women and carriers of germline BRCA and PALB2 mutations are disproportionately affected by triple-negative breast malignancies. The risk of death from TNBC is still almost twice as high for African Americans with the disease, even after adjusting for socioeconomic status, stage, and delays in treatment (9). Epidemiologic research, such as the Carolina Breast Cancer Study, indicates that basal-like tumors, compared to luminal A tumors, are more prevalent among women with early menarche, higher parity, younger age at full-term pregnancy, shorter breastfeeding duration, higher body mass index, and higher waist-to-hip ratio, particularly in premenopausal patients (10). Additionally, Bauer et al. identified that younger, non-Hispanic Black and Hispanic women with TNBC exhibit more aggressive tumors and poorer survival rates, regardless of stage, with non-Hispanic Black women with late-stage TNBC having the lowest survival rates (11).

In comparison to the luminal subtype, several studies have consistently demonstrated that basal-like BC, including TNBC, has a worse prognosis and worse BC-specific survival (10, 12). In comparison to individuals without TNBC, those with TNBC had a greater risk of mortality and distant recurrence, according to a recent Canadian study including over 1,500 women (6). Research has consistently shown that, in contrast to ER-positive BC, TNBC is linked to more aggressive relapses in visceral and soft tissues, while relapses in the bone are less frequent (13).

A retrospective study of 33,654 female patients with TNBC identified key prognostic factors and highlighted the benefits of primary tumor surgery. Younger age, white race, married status, lower tumor grade and stage, and undergoing surgery were associated with better outcomes. A predictive nomogram developed from the study demonstrated high accuracy. Notably, surgery improved cancer-specific survival across all risk groups, suggesting its potential benefit in TNBC treatment strategies (14).

### 1.3 Current treatment strategies

TNBC is an aggressive subtype distinguished by the absence of ER, PR, and HER2 expression, limiting the availability of targeted therapy and making chemotherapy the primary therapeutic option (15).

TNBC usually responds effectively to chemotherapy (16), especially in neoadjuvant or adjuvant settings, with anthracycline- and taxane-based regimens accomplishing pathological complete response (pCR) in 30-40% of early-stage cases (17–19). Anthracyclines such as doxorubicin and epirubicin cause DNA damage and apoptosis (20–23) but are associated with cardiotoxicity (24–26); however, epirubicin has a better toxicity profile (27–29). Taxanes, such as paclitaxel and docetaxel, disrupt mitosis by stabilizing microtubules and are more effective when coupled with platinum drugs, however resistance may develop in BRCA1-mutated cancers (30). Platinum compounds, such as carboplatin, form DNA cross-links that cause apoptosis and are particularly beneficial when combined with neoadjuvant regimens, as seen in studies such as KEYNOTE-522 and BrighTNess (28–35). These drugs improve pCR and event-free survival (EFS). In the KEYNOTE-522 trial, the addition of pembrolizumab to neoadjuvant chemotherapy resulted in a significantly higher pCR rate—64.8% versus 51.2% with chemotherapy alone—representing a 13.6% absolute increase (p < 0.001). This benefit translated into improved long-term outcomes, with an estimated overall survival (OS) at 60 months of 86.6% (95% confidence interval [CI], 84.0 to 88.8) in the pembrolizumab–chemotherapy group, compared with 81.7% (95% CI, 77.5 to 85.2) in the placebo–chemotherapy group (p = 0.002) (36).

Radiotherapy is indicated in TNBC patients of all ages who have undergone lumpectomy and/or present with positive axillary lymph node evaluation, as it improves locoregional control and overall survival (OS) (37–42).

Targeted strategies are transforming the TNBC therapy landscape (43), beginning with PARP inhibitors such as olaparib and talazoparib, which exploit homologous recombination repair deficits in BRCA-mutated cancers, resulting in tumor cell death (44–52). Synthetic lethality is a concept in which the simultaneous disruption of two genes leads to cell death, whereas the loss of either gene alone is tolerated. This approach offers a targeted therapeutic strategy in TNBC, by exploiting specific genetic vulnerabilities - such as BRCA mutations - using PARP inhibitors. While successful in BRCA-mutated TNBC, these drugs have limited efficacy in BRCA wild-type instances and may experience resistance, urging the investigation of combination strategies with agents such as carboplatin. CDK4/6 inhibitors, which have been beneficial in HR-positive malignancies, have the potential to treat TNBC's luminal androgen receptor subtype by targeting abnormal cell cycle progression (53–56). Similarly, many TNBCs, particularly the basal-like subtype, overexpress EGFR, which is a target for tyrosine kinase inhibitors like gefitinib and monoclonal antibodies like cetuximab (57). Although dual inhibition strategies have proven ineffective, combining EGFR-targeted medicines with platinum chemotherapy has increased survival rates (58–63). However, these targeted approaches are currently not recommended by major clinical guidelines, including ESMO and NCCN, outside of clinical trials.

Given TNBC's high programmed cell death ligand 1 (PD-L1) expression and abundance of tumor-infiltrating lymphocytes (TILs), immunotherapy has grown in importance (64–70). Immune checkpoint inhibitors (ICIs), such as pembrolizumab, have improved outcomes when combined with chemotherapy, as shown in trials like KEYNOTE-522 —where PD-L1 status is not a criterion for use— leading to FDA approval for pembrolizumab in high-risk, early-stage, TNBC (71). These treatments increase the risk of immune-related side effects, especially during the neoadjuvant phase. Combination regimens that combine ICIs with DNA repair inhibitors such as PARP inhibitors have demonstrated synergistic effects, boosting anti-tumor immunity, particularly in homologous recombination-deficient cancers (72–77). However, the combination of the two drugs is not yet in clinical practice. Antibody-drug conjugates (ADCs), such as sacituzumab govitecan, which targets Trop-2, provides strong cytotoxins directly to tumor cells and have shown efficacy in both metastatic and neoadjuvant settings, providing prolonged survival with controlled toxicity (78–80).

Despite these developments, TNBC is still challenging to treat due to its heterogeneity, lack of prognostic biomarkers, and high recurrence rate. Continued research is required to maximize the use of innovative drugs, uncover biomarkers for patient stratification, and devise reasonable combination methods that improve efficacy while limiting toxicity.

The combination of immunotherapy, targeted medicines, and ADCs broadens the therapeutic arsenal and has the potential for more tailored, long-lasting treatment outcomes in TNBC.

## 2 Neoadjuvant chemo-immunotherapy in TNBC

### 2.1 Rationale for neoadjuvant therapy

Neoadjuvant therapy in BC pertains to the administration of systemic therapy before the surgical intervention. The treatment aims to downstage and downsize, to reduce the size of unresectable tumors, to allow conservative surgeries and to address micrometastases. Neoadjuvant chemotherapy (NAC) has gained significant acceptance as the established therapeutic approach for early TNBC to proactively anticipate tumor response and administer appropriate adjuvant therapies (81). Multiple studies have shown that TNBC exhibits significantly higher pCR rates following NAC compared to hormone receptor-positive BC, with pCR being strongly associated with better outcomes. A large study from the MD Anderson Cancer Center, involving 1,118 patients treated between 1985 and 2004, found that the pCR rate in TNBC was double that of non-TNBC (22% vs. 11%). Despite this, TNBC patients had poorer 3-year PFS and OS rates compared to non-TNBC patients. However, TNBC patients who achieved a pCR had similar 3-year OS rates to non-TNBC patients (94% vs. 98%), while those with residual disease after treatment had significantly worse outcomes. These findings highlight the importance of achieving pCR in improving long-term survival for TNBC patients, as those with residual disease face a higher risk of recurrence and death (82).

In addition, the pooled analysis by Cortazar and Geyer (83), which included 11,955 patients across 12 international neoadjuvant trials, reinforced the prognostic value of pCR in breast cancer. The study demonstrated that achieving pCR was strongly associated with improved event-free survival and overall survival, particularly in aggressive subtypes such as TNBC and HER2-positive tumors, whereas its prognostic significance was limited in luminal A disease. Importantly, this analysis provided large-scale validation that pCR can serve as a surrogate endpoint for long-term outcomes in clinical trials, highlighting its utility as both a prognostic biomarker and a measure of therapeutic efficacy in TNBC.

Neoadjuvant chemotherapy is the recommended method for treating stage II or III TNBC. In the context of early-stage high-risk TNBC, the inclusion of pembrolizumab in taxane platinum-based chemotherapy, followed by an anthracycline, resulted in a notable enhancement in pCR rates and an improvement in event-free survival (EFS) (84). In KEYNOTE-522, pembrolizumab was incorporated not only during the taxane/platinum phase but also in the subsequent anthracycline–cyclophosphamide regimen, underscoring its integration across the entire neoadjuvant treatment course.

In a recent comprehensive meta-analysis, von Minckwitz et al. analyzed data from 6,377 patients with operable or locally advanced, non-metastatic BC treated with neoadjuvant anthracyclines and taxanes, with or without trastuzumab (18). The objective of this study was to validate different definitions of pCR and to evaluate its prognostic significance in terms of disease-free survival (DFS) and OS across various BC subtypes. The study concluded that pCR should be conservatively defined as ypT0 ypN0, excluding ductal carcinoma *in situ*. Furthermore, pCR was found to be a reliable surrogate marker for survival in patients with luminal B (HER2-negative), HER2-positive (non-luminal), and TNBC subtypes, but not for those with luminal A or luminal B/HER2-positive subtypes.

Additionally, Huober et al. observed that TNBC tumors often demonstrate a rapid response to neoadjuvant therapy, with significant tumor reduction evident after only two treatment cycles (85). This finding highlights the critical importance of neoadjuvant chemotherapy in treatment of TNBC.

### 2.2 Neoadjuvant chemotherapy and immunotherapy strategies: current evidence and clinical trials

Despite advances in targeted and biological therapies, cytotoxic chemotherapy remains the cornerstone of neoadjuvant treatment for TNBC (86). Anthracycline/taxane-based regimens are widely employed and supported by retrospective analyses and subgroup data from trials predating 2010 (87). For instance, anthracyclines alone have yielded pCR rates between 14–47%, while sequential regimens combining anthracyclines with taxanes report pCR rates ranging from 17–39% (88–92). Notably, the GeparTrio trial reported pCR rates up to 57% with neoadjuvant anthracyclines, cyclophosphamide, and taxanes (85).

To enhance these outcomes, numerous strategies have been pursued to increase pCR rates, which serve as an intermediate surrogate for improved long-term survival. These include the incorporation of DNA-damaging agents, particularly platinum compounds like carboplatin, given the DNA repair deficiencies frequently found in TNBC. Several clinical trials have assessed this approach (46, 93, 94). The GeparSixto trial showed a significant improvement in pCR rates from 36.9% to 53.2% when carboplatin was added, albeit with increased hematologic toxicity (46, 93, 94). Similarly, the BrighTNess trial demonstrated that the observed improvements in pCR and event-free survival (EFS) were attributable to carboplatin rather than the PARP inhibitor veliparib (95). In contrast, CALGB 40603, which also evaluated the addition of carboplatin to a standard AC-T regimen, did not find a statistically significant improvement in EFS despite a numerically higher pCR (96). Collectively, these findings support the use of platinum-based regimens in selected patients, particularly those with stage II–III TNBC, while highlighting the need to balance efficacy with tolerability.

Beyond chemotherapy, immunotherapy has recently emerged as a pivotal component in the neoadjuvant management of TNBC (71), with the rationale rooted in the immunogenic potential of this subtype. Standard chemotherapeutics can modulate the tumor microenvironment (TME), enhancing antigen presentation, promoting T cell infiltration (97), and reducing immunosuppression (98). This primes the immune system for checkpoint inhibition and supports the integration of ICIs with chemotherapy.

The KEYNOTE-522 trial exemplifies this strategy. In this phase III study, pembrolizumab—a PD-1 inhibitor—was added to a neoadjuvant regimen of carboplatin, paclitaxel, anthracycline, and cyclophosphamide in patients with stage II–III TNBC (71, 71, 84). The trial demonstrated a statistically significant increase in pCR rates, from 51.2% in the placebo group to 64.8% in the pembrolizumab group (a 13.6% absolute increase; p < 0.001). Furthermore, EFS improved by 7% at 3 years, with a hazard ratio of 0.63 (95% CI 0.48–0.82). These benefits were observed regardless of PD-L1 status, suggesting that PD-L1 may hold prognostic but not predictive value in early TNBC. Importantly, patients with residual disease after neoadjuvant therapy also derived benefit from continued pembrolizumab in the adjuvant setting, further validating this approach as a new standard of care for high-risk TNBC.

Other notable trials have also explored the role of ICIs in the neoadjuvant setting (**Table 1**). The IMpassion031 trial investigated atezolizumab (anti–PD-L1) with chemotherapy and showed an increased pCR rate but no EFS benefit (99). The NeoTRIP trial evaluated atezolizumab combined with carboplatin and nab-paclitaxel in high-risk early TNBC. While pCR rates were not significantly improved with atezolizumab versus chemotherapy alone (43.5% vs 40.8%, OR 1.11, 95% CI 0.69–1.79), updated analyses suggested a trend toward improved 5-year event-free survival (62% vs 57%; HR 0.81, 95% CI 0.52–1.27), although not statistically significant (100). By contrast, GeparNuevo assessed durvalumab in combination with chemotherapy and, while the increase in pCR was modest and not statistically significant (53.4% vs 44.2%; Δ 9.2%, p=0.287), long-term follow-up revealed a marked survival advantage. At 3 years, durvalumab significantly improved invasive disease–free survival (iDFS; HR 0.48, 95% CI 0.24–0.97), distant disease–free survival (DDFS; HR 0.46, 95% CI 0.23–0.92), and OS (HR 0.24, 95% CI 0.08–0.72), underscoring that survival gains may emerge independently of pCR improvements (101). These discrepancies underscore the importance of trial design, backbone chemotherapy regimens, and biomarker selection when interpreting ICI efficacy in early TNBC.

**Table 1:** Neoadjuvant chemoimmunotherapy clinical trials and efficacy in triple-negative breast cancer.

Trial name	Phase	Intervention	Comparator	Primary outcome	Results summary
KEYNOTE-522	III	Pembrolizumab + chemotherapy	Placebo + chemotherapy	pCR rate	pCR: 64.8% in pembrolizumab group vs. 51.2% in placebo group
GeparNuevo	II	Durvalumab + chemotherapy	Placebo + chemotherapy	pCR rate	pCR: 53.4% in durvalumab group vs. 44.2% in placebo group
NeoTRIPaPDL1	III	Atezolizumab + nab-paclitaxel	Placebo + nab-paclitaxel	EFS	No significant difference in pCR rates between groups
IMpassion031	III	Atezolizumab + chemotherapy	Placebo + chemotherapy	pCR rate	pCR: 57.6% in atezolizumab group vs. 41.1% in placebo group
BrightTNess	III	Veliparib + carboplatin + standard chemotherapy	Standard chemotherapy alone	pCR rate	pCR: 53% in veliparib group vs. 31% in control group
CALGB 40603	II	Chemotherapy + carboplatin ± bevacizumab	Standard chemotherapy	pCR rate	Both carboplatin and bevacizumab improved pCR rates

EFS, event-free survival, pCR , complete pathologic response.

The NeoSTAR Arm A2 trial (102) evaluated neoadjuvant sacituzumab govitecan (SG) combined with pembrolizumab in 50 patients with early-stage TNBC. Patients received SG (10 mg/kg, days 1 and 8) plus pembrolizumab (200 mg, day 1) for four 21-day cycles. The pCR rate after SG/pembrolizumab alone was 34%, increasing to 50% overall after additional neoadjuvant chemotherapy. Response rate (CR + PR) was 66%. Among five patients with pathogenic BRCA mutations, 60% achieved pCR with SG/pembrolizumab. The regimen was generally well tolerated; grade ≥3 adverse events occurred in 40%, mainly nausea, alopecia, fatigue, and diarrhea. These results support further investigation of ADC–ICI combinations in early TNBC.

In conclusion, the integration of platinum agents and immunotherapy into standard neoadjuvant regimens has reshaped the therapeutic landscape of TNBC. Carboplatin offers a modest but meaningful increase in pCR, particularly in BRCA-deficient or homologous recombination-deficient tumors. The addition of pembrolizumab, as demonstrated in KEYNOTE-522, represents a major advancement, combining improved pCR and EFS without reliance on PD-L1 expression. These findings strongly support chemoimmunotherapy as the new standard in the neoadjuvant treatment of early-stage TNBC.

## 3 Biomarker-based immune monitoring strategies

**Figure 2:**
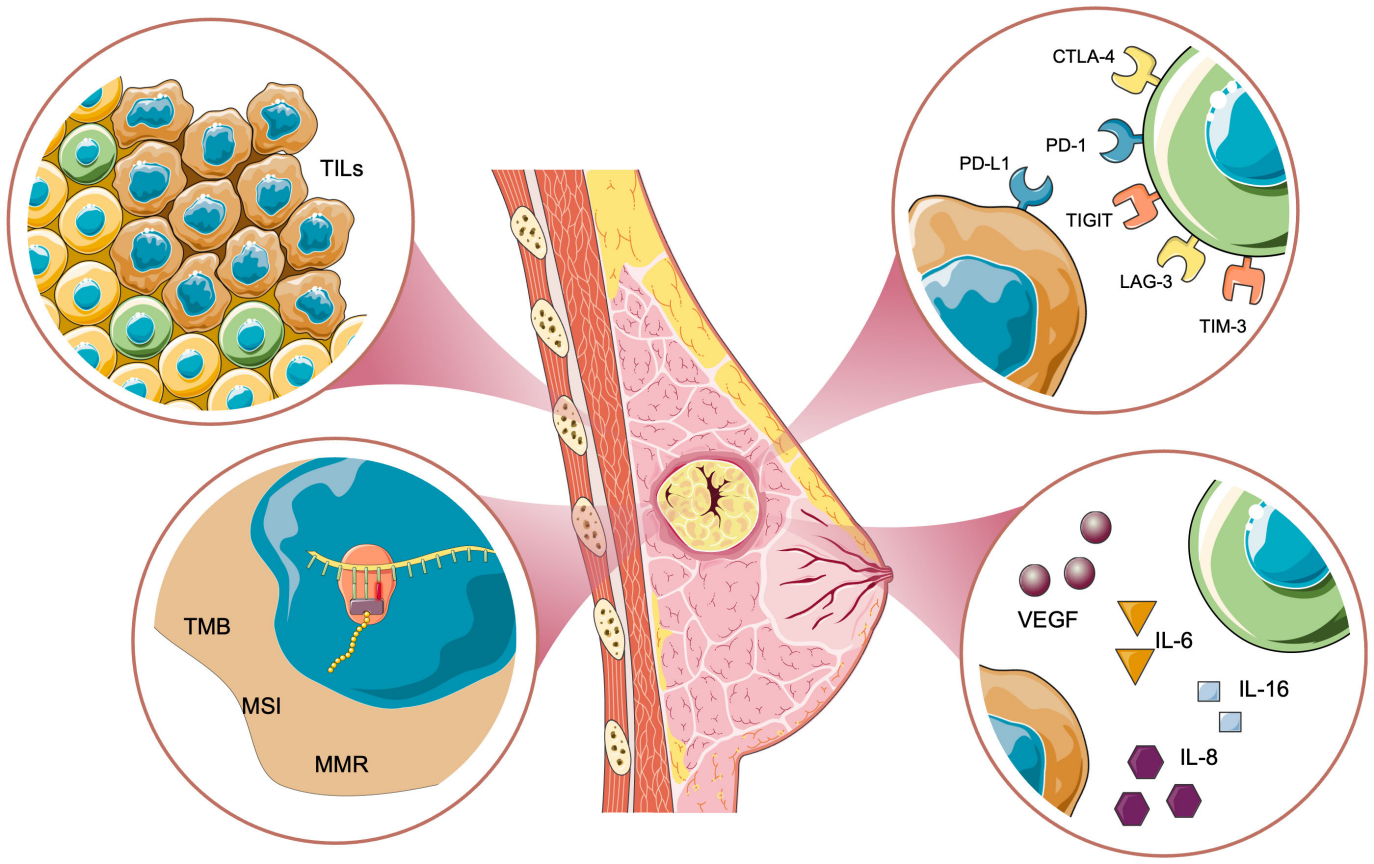
Immune response in triple-negative breast cancer. This schematic illustrates key biomarkers and immune mechanisms in triple-negative breast cancer (TNBC). Tumor-infiltrating lymphocytes (TILs) represent immune activity within the tumor microenvironment. Genomic biomarkers include tumor mutational burden (TMB), microsatellite instability (MSI), and mismatch repair deficiency (MMR), which contribute to tumor progression and immune recognition. Cytokines and soluble factors such as VEGF, IL-6, and IL-8 promote angiogenesis and immune suppression. Immune checkpoint pathways, including PD-L1/PD-1, CTLA-4/B7, and TIGIT, LAG3, TIM3 interactions, mediate immune evasion.

A comprehensive understanding of the tumor-immune interface in TNBC requires the integration of diverse biomarker classes that span tissues, blood, imaging, and dynamic temporal signatures. Tissue-based biomarkers have historically served as the foundation for assessing and predicting immunotherapy response. These include well-established targets such as PD-L1, microsatellite instability (MSI), tumor mutational burden (TMB), and various tumor gene expression signatures (**Figure 2**).

Together, they provide a valuable framework for evaluating tumor immunogenicity and potential responsiveness to immune checkpoint blockade (ICB) therapies (103–105). Their relevance in neoadjuvant chemo-immunotherapy for TNBC is rapidly evolving as we deepen our understanding of tumor-immune interactions in this particularly aggressive BC subtype (36, 106).

**Figure 3:**
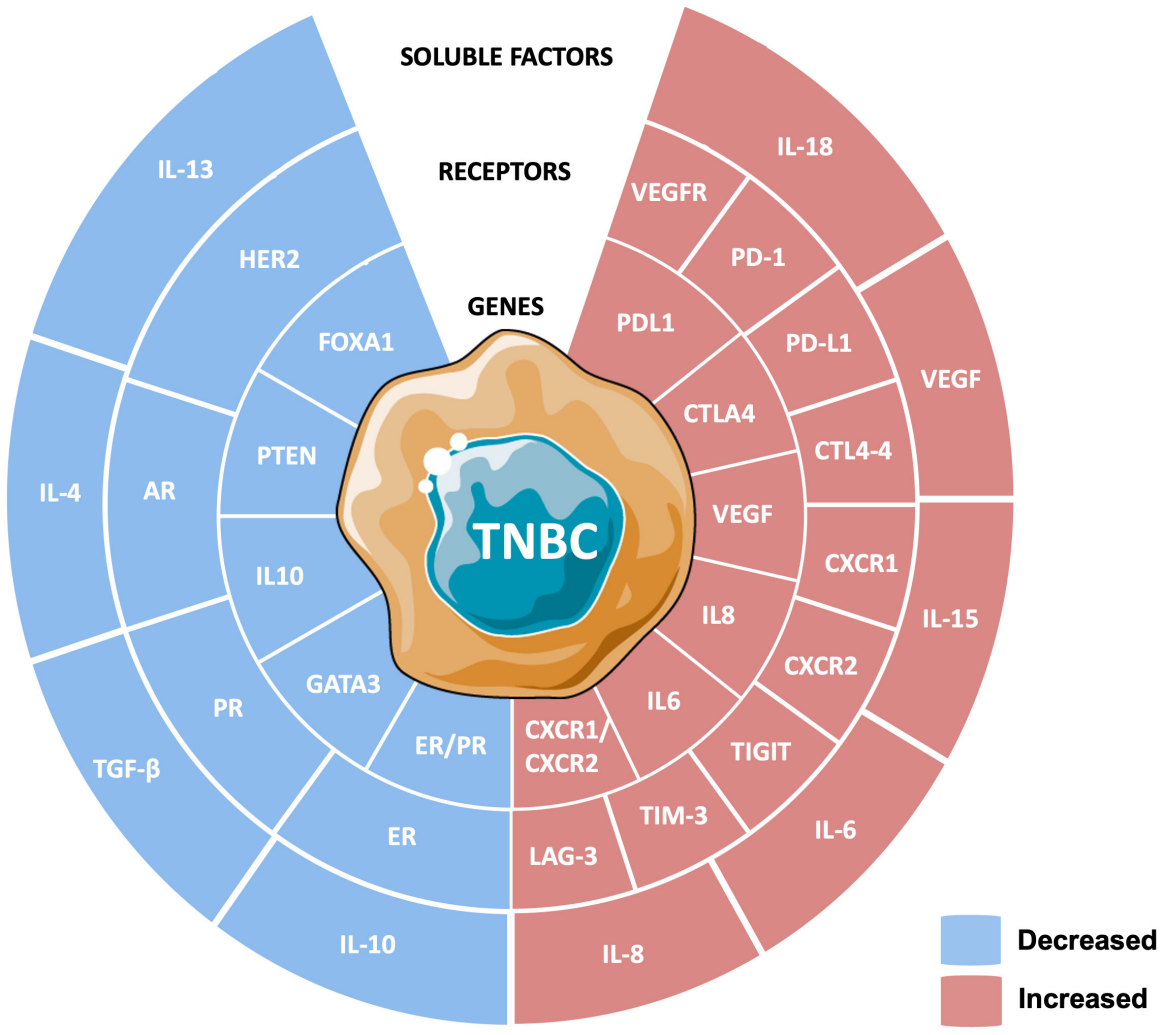
Biomarker profile of triple-negative breast cancer (TNBC). Dysregulated biomarkers in TNBC, classified into soluble factors, receptors, and genes. Blue regions represent decreased biomarkers, including IL-4, IL-10, TGF-β, HER2, ER, PR, FOXA1, GATA3, and PTEN, signifying the loss of hormone receptor expression and tumor-suppressive pathways. Red regions depict increased biomarkers such as pro-inflammatory cytokines (IL-6, IL-8, IL-18), angiogenic factor VEGF, immune checkpoints (PD-1, PD-L1, CTLA-4, TIGIT, LAG-3, TIM-3), and chemokine receptors (CXCR1, CXCR2).

The tumor microenvironment plays a crucial role in progression, influenced by a balance of immune-suppressive and immune-activating factors. The illustration in **Figure 3** captures the complexity of TNBC by showcasing key genes, receptors, and soluble factors that shape immune responses.

### 3.1 Tissue-based biomarkers

#### 3.1.1 Programmed death-ligand 1

PD-1 is a critical immune checkpoint receptor that plays a central role in regulating immune responses and maintaining self-tolerance by limiting T-cell activity in peripheral tissues. PD-1 binds two ligands: PD-L1 and PD-L2, which have distinct expression profiles across various tumor types. PD-L1 expression is regulated by several mechanisms, including oncogenic signaling pathways, loss or silencing of PTEN, and activation of the PI3K pathway (107, 108). Monoclonal antibodies targeting PD-1 or PD-L1 can block this inhibitory axis, thereby reactivating anti-tumor immune responses by lifting T-cell suppression.

PD-L1 is among the most extensively studied biomarkers in cancer immunotherapy and has gained regulatory approval to guide the use of PD-1/PD-L1 inhibitors in multiple malignancies (109, 110). In clinical settings, PD-L1 expression is most assessed using immunohistochemistry (IHC) and is quantified using the Tumor Proportion Score (TPS) or the Combined Positive Score (CPS) (111). While TPS evaluates the proportion of PD-L1-positive tumor cells, CPS incorporates both tumor and immune cell staining to provide a broader measure of immune engagement. Higher TPS and CPS scores have been correlated with improved responses to anti-PD-1/PD-L1 therapies in various cancers, including non-small cell lung cancer (109, 110).

In TNBC, the role of PD-L1 as a biomarker is more nuanced. PD-L1 expression has been detected on both tumor and immune cells in TNBC, but its assessment is complicated by spatial heterogeneity and temporal dynamics of expression (111). Studies have shown that PD-L1 levels can vary depending on tumor stage (primary vs. metastatic), site of disease, and technical variables such as antibody clones and scoring thresholds (112). Despite this complexity, PD-L1 remains an active focus of investigation in TNBC, particularly as a candidate biomarker for patient stratification in neoadjuvant immunotherapy trials.

Checkpoint inhibitors targeting PD-1/PD-L1 have shown promising efficacy in early-stage TNBC. In this context, the KEYNOTE-522 trial was the first phase III study to demonstrate a significant improvement in clinical outcomes with the addition of immunotherapy. Patients receiving neoadjuvant chemotherapy plus pembrolizumab showed a 13.6% absolute increase in pCR compared to those receiving chemotherapy alone (64.8% vs. 51.2%) (71). Notably, this benefit was observed regardless of PD-L1 status, indicating that PD-L1 expression may not be a definitive predictor of response in early TNBC (85). This finding aligns with data from phase II trials showing that tumors lacking PD-L1 expression can still respond to immune checkpoint blockade (88). Nevertheless, PD-L1 positivity has been associated with favorable prognostic indicators such as higher pCR rates, improved metastasis-free survival, and OS in TNBC (101, 111, 112).

#### 3.1.2 Microsatellite instability

Another critical tissue-based biomarker is microsatellite instability (MSI), which reflects defects in the DNA mismatch repair (MMR) machinery. Tumors with high levels of MSI (MSI-H) typically harbor elevated mutational loads and generate abundant neoantigens, enhancing their visibility to the immune system. MSI-H is very rare in breast cancer, with reported prevalence of approximately 0.6–2% (113, 114). Nevertheless, MSI-H status has been associated with robust responses to PD-1 blockade, as shown in the KEYNOTE-158 trial, which led to FDA approval of pembrolizumab for MSI-H/dMMR cancers regardless of tumor origin (128–120) (115).

A study investigated mismatch repair and microsatellite instability in TNBC by analyzing tissue samples from 440 patients. The results showed that mismatch repair deficiency was rare, with only one sample identified as deficient and no cases of high-frequency microsatellite instability. Most samples had either no microsatellite instability or low-frequency instability. Additionally, no significant correlations were found between mismatch repair or microsatellite instability status and clinicopathological features, PD-1/PD-L1 expression, or survival. The findings suggest that mismatch repair and microsatellite instability are infrequent in TNBC and may have limited prognostic value, highlighting the need for further research into alternative biomarkers for immunotherapy (116).

Nonetheless, its conceptual importance lies in establishing a mechanistic link between genomic instability and immune sensitivity, a principle that continues to inform biomarker discovery in BC and beyond (116).

#### 3.1.3 Tumor mutational burden

TMB represents another biomarker of interest, defined as the number of non-synonymous somatic mutations per megabase of coding DNA. A high TMB is thought to correlate with increased neoantigen presentation and subsequent T-cell priming, thereby enhancing susceptibility to immunotherapy (117, 118). However, there is a scarcity of data on the actual therapeutic significance of this biomarker in BC settings (119).

In BC, the predictive value of TMB remains controversial due to limited data. While high TMB is observed in up to 3% of primary BC tumors and 11% of metastatic cases, these tumors show greater sensitivity to checkpoint inhibitors. However, high TMB has not been associated with improved OS in BC patients receiving immunotherapy (120, 121). In June 2020, the FDA granted approval for pembrolizumab in cases of TMB high (≥10 mut/Mb), unresectable, or metastatic cancer (122).

Evidence from the KEYNOTE-158 trial has demonstrated improved objective response rates (ORR) and PFS in patients with high TMB across multiple tumor types (123). While data specific to TNBC remain limited, the general principles of immunogenicity conferred by high TMB are being explored in ongoing BC trials. Importantly, TMB should not be viewed in isolation. Rather, it is increasingly clear that integrating TMB with other immunological parameters—such as immune infiltrate composition, T-cell receptor clonality, and expression of immune modulatory genes—can provide a more comprehensive and accurate prediction of therapeutic outcomes (124).

#### 3.1.4 Tumor-infiltrating lymphocytes

Tumor-infiltrating lymphocytes (TILs) are a heterogeneous population of immune cells, mainly composed of T cells, with smaller fractions of B cells and natural killer (NK) cells, that localize in the tumor microenvironment (125). TILs are present either in the tumor stroma or directly within tumor cell nests, and they play a central role in mediating antitumor immunity. Their infiltration reflects the immunogenicity of the tumor and the host immune response. In addition to general lymphocyte populations, immune regulators such as FOXP3+ regulatory T cells (Tregs), TIM3, LAG3, and TIGIT are often expressed on TILs. LAG3 is an inhibitory immune checkpoint receptor expressed on activated T cells, NK cells, B cells, and plasmacytoid dendritic cells (pDCs), and binds to MHC class II molecules. In TNBC mouse models, dual blockade of LAG3 and PD-1 elicits a more potent anti-tumor effect than monotherapy. TIM3, another checkpoint molecule, is expressed on Tregs, dendritic cells, and subsets of lymphocytes and monocytes (126).

TILs have been broadly recognized as prognostic and predictive biomarkers in cancer. Their prevalence varies among BC subtypes, with TNBC and HER2+ subtypes showing higher TIL infiltration compared to luminal-like tumors (127). In the broader oncology setting, higher TIL levels have been correlated with improved response to chemotherapy and immunotherapy, reduced recurrence, and longer survival. Tumors with abundant TILs are often classified as “hot” or inflamed and are more likely to respond to ICIs (128). Interestingly, aggressive BC subtypes with poor prognosis—such as ER-negative, PR-negative, or node-positive tumors—also tend to have elevated TIL levels (128, 129).

In TNBC specifically, TILs serve as key indicators of tumor immune microenvironmental activity. Their abundance is associated with reduced proliferation, metastasis, and therapy resistance (16). Salgado et al. reported that intratumoral lymphocytes significantly predicted pCR to neoadjuvant chemotherapy in both training and validation cohorts (P = 0.012 and P = 0.001) (130). In lymphocyte-predominant breast cancers, pCR rates reached 40–42%, versus only 3–7% in TIL-low tumors. Adams et al. confirmed that higher stromal TIL (sTIL) scores were linked to better prognosis and lower recurrence in two national clinical trials involving 481 TNBC patients (131). Moreover, PD-L1 expression on TILs—rather than on tumor cells—has been linked to improved clinical outcomes, likely reflecting adaptive immune activation. Sugie et al. showed that PD-L1 on immune cells strongly correlates with CD8+ T-cell infiltration and total TIL content in TNBC, supporting its role as a surrogate marker of T-cell–inflamed tumors (130, 132).

Evidence from large, pooled analyses has further strengthened the prognostic and predictive role of TILs. Denkert et al. (97) analyzed data from over 2,500 early-stage TNBC patients across multiple neoadjuvant trials and confirmed that each 10% increase in sTILs was associated with a proportional increase in pCR rates and improved DFS and OS. Similarly, Loi et al. (133) performed a pooled analysis of TNBC cohorts from clinical trials and demonstrated that high sTILs consistently predicted improved outcomes, including OS, independent of chemotherapy regimen or nodal status. These large-scale data underscore that TIL quantification is a robust biomarker for treatment planning and risk stratification.

Clinical trial data further demonstrate the impact of TILs on immunotherapy efficacy. In the phase III IMpassion130 trial, atezolizumab combined with nab-paclitaxel led to prolonged progression-free survival (PFS) in patients with tumors harboring ≥10% stromal TILs, with greater benefit observed in PD-L1–positive cases (134). Furthermore, higher proportions of CD8+ TILs have been associated with improved responses to ICIs in TNBC patients (135, 136). Elevated TIL levels in TNBC also correlate with a greater likelihood of pCR to chemotherapy—a surrogate marker of long-term survival (137). Conversely, low TIL infiltration or absence of immune markers may signal poor responsiveness, necessitating alternative therapeutic strategies.

While most studies indicate improved outcomes in patients with elevated sTILs, a few have noted that post-chemotherapy TIL enrichment may signal relapse risk in certain TNBC subgroups (138). Nevertheless, the bulk of evidence—including several large prognostic analyses—demonstrates that higher sTIL levels consistently predict better short- and long-term outcomes (139, 140). These beneficial effects are largely attributed to the presence of CD4+ and CD8+ effector T cells, which mediate anti-tumor activity and correlate with immunotherapy benefit (140, 141).

#### 3.1.5 Gene expression signatures

In addition to PD-L1, MSI, and TMB, gene expression signatures derived from tumor transcriptomes are emerging as powerful tools for immune monitoring. Among these, interferon-gamma (IFN-γ)-associated signatures have garnered significant attention. IFN-γ is a key effector cytokine produced by activated T cells and NK cells, and its downstream signaling orchestrates a cascade of immune-stimulatory effects, including upregulation of MHC molecules, chemokine production, and antigen presentation machinery (142). In melanoma, IFN-γ gene signatures have been successfully used to stratify patients in neoadjuvant immunotherapy trials, such as the DONIMI study, where they predicted response to PD-1 and CTLA-4 blockade (143). In breast cancer, Heimes et al. (144) reported that high IFN-γ expression correlated with improved disease-free and overall survival, supporting its role as a prognostic biomarker. Although less studied in TNBC, these signatures offer a promising avenue for selecting patients most likely to benefit from neoadjuvant immune-based therapies, particularly in immunologically “cold” tumors that may require priming or combination strategies to initiate effective immune responses.

### 3.2 Blood-based biomarkers

Beyond tumor tissues, peripheral blood offers a non-invasive window into the systemic immune state of cancer patients. Liquid biopsies can be repeatedly sampled over time, enabling longitudinal monitoring of treatment effects and immune dynamics. Among the blood-based biomarkers, soluble immune proteome profiling stands out for its ability to capture real-time fluctuations in cytokines, chemokines, and soluble checkpoint molecules. These circulating analytes provide insight into the activation status, regulatory balance, and effector function of the immune system throughout the course of therapy. For example, elevated levels of pro-inflammatory cytokines such as IL-6, TNF-α, or IFN-γ may indicate an active anti-tumor immune response or, conversely, immune-related toxicity (145, 146).

#### 3.2.1 Immune-related gene expression profiling

Another informative blood-based modality is immune-related gene (IRG) expression profiling. By assessing mRNA transcripts in peripheral blood mononuclear cells (PBMCs) or circulating tumor cells (CTCs), researchers can monitor immune activation or suppression in response to therapy. This technique allows for the detection of gene expression patterns associated with cytotoxicity, antigen presentation, T-cell exhaustion, and regulatory immune networks (105).

#### 3.2.2 Comprehensive immunophenotyping

Comprehensive immunophenotyping plays a critical role in monitoring immune responses during neoadjuvant chemo-immunotherapy for TNBC, providing detailed insights into immune cell dynamics in both the peripheral blood and tumor microenvironment. Using advanced technologies such as multiparametric flow cytometry and mass cytometry (CyTOF), researchers can analyze dozens of markers at the single-cell level to assess cell lineage, activation status, differentiation, and exhaustion (147). In TNBC, treatment response has been associated with increased frequencies of activated effector CD8^+^ T cells and decreased regulatory T cells (Tregs), as well as modulation of myeloid populations and antigen-presenting cells (111). Wang et al. (148) demonstrated that longitudinal single-cell profiling can identify early immune signatures predictive of pathological complete response, including expansion of specific cytotoxic T-cell subsets and reduction of immunosuppressive myeloid populations. These phenotypic changes reflect the evolving tumor–immune interaction under therapeutic pressure. Longitudinal immunophenotyping allows for temporal tracking of immune reconstitution and may reveal early predictors of pCR (147). Integration with transcriptomics or spatial imaging further enhances biomarker discovery. Integration with transcriptomics or spatial imaging further enhances biomarker discovery. As TNBC is highly immunologically heterogeneous, immunophenotyping is indispensable for personalizing treatment and informing early decision-making in clinical trials. Thus, comprehensive immune profiling supports both mechanistic understanding and translational implementation of immunotherapy in TNBC.

#### 3.2.3 Cytokine profiling

Inflammation is essential for the immune system's defense against pathogens like viruses and bacteria, and it is also involved in tumor development, angiogenesis, and metastasis (149). Oncogenic changes, hypoxia, cytokines, and chemokines attract inflammatory cells to the tumor microenvironment, which includes immune cells and activated fibroblasts that secrete factors promoting tumor growth (150). Recent research highlights that inflammation signaling, particularly through the IL-6/JAK2/Stat3 pathway, is significant in maintaining the stem cell-like properties of BC (151). Although most studies have reported higher cytokine levels in BC patients, one study involving 90 BC patients and 15 healthy volunteers found no significant difference in baseline cytokine levels between the two groups, as indicated by plasma concentrations of IL-1β, IL-6, IL-8, IL-10, IL-12, and TNF-α. However, a direct link between inflammatory cytokine levels and clinical outcomes in BC patients has not been clearly established (152). Angiogenesis, driven by factors like vascular endothelial growth factor (VEGF), is crucial for cancer growth and metastasis. VEGF, particularly elevated in TNBC, not only promotes tumor progression but also serves as a key target in anti-angiogenic therapies, improving survival rates when combined with chemotherapy, as demonstrated in metastatic colorectal cancer (153). Beyond chemotherapy, combining VEGF inhibition with immunotherapy has shown promise in other malignancies. In advanced endometrial carcinoma, the combination of lenvatinib (a VEGF receptor inhibitor) and pembrolizumab led to significantly longer PFS and OS compared to chemotherapy alone (154). Similarly, in clear cell renal cell carcinoma, combining VEGF inhibition with ICIs has demonstrated improved outcomes (155). These findings suggest that integrating VEGF-targeted therapies with immunotherapy could enhance treatment efficacy in various cancers. The significance of VEGF in TNBC is underscored by its notably higher expression levels three times greater than in ER/PR-positive tumors and 1.5 times higher than in HER2-positive tumors—making it a critical biomarker for prognosis and treatment (156–158). Similarly, IL-8 is highly expressed in the stroma of TNBC, significantly contributing to tumor growth and metastasis by promoting angiogenesis, proliferation, and migration of tumor cells. The interaction of IL-8 with its receptors, CXCR1 and CXCR2, is crucial for TNBC progression, making these molecules promising therapeutic targets. Studies demonstrate that blocking the IL-8 signaling pathway can effectively reduce TNBC cell proliferation and migration, highlighting its importance in developing treatment strategies (159, 160). In addition to IL-8, other cytokines like IL-15 and IL-18 play pivotal roles in the tumor microenvironment. IL-15 shows potential in enhancing NK cell-mediated anti-tumor activity (161, 162), while IL-18 is associated with poor survival outcomes due to its role in increasing immunosuppressive NK cells and upregulating PD-1 expression. Together, these cytokines underscore the complexity of the TNBC microenvironment (163, 164).

In parallel, recent research has explored blood cytokine profiles in TNBC patients to identify biomarkers that could predict treatment outcomes. This study identified five cytokines—IL-1α, TRAIL, SCF, CCL5, and IL-16—linked to favorable clinical outcomes, with their levels decreasing during chemotherapy and rebounding afterward. Notably, patients with consistently high levels of these cytokines throughout treatment experienced better outcomes. These findings suggest that monitoring cytokine levels over time could enhance the precision of TNBC treatment, paving the way for more personalized therapeutic approaches (165).

The data regarding immune response biomarkers in TNBC is summarized in **Table 2**.

**Table 2:** Immune response biomarkers in cancer immunotherapy.

Biomarker	Role	Clinical significance	Predictive/prognostic value
PD-L1	Immune checkpoint ligand inhibiting T-cell activity.	Associated with response to immune checkpoint inhibitors.	Linked to improved survival; may not predict response in early-stage TNBC.
CTLA-4	Inhibits T-cell activation, maintaining immune homeostasis.	High expression correlates with metastasis.	Elevated expression in 50% of breast tumors, associated with poor prognosis.
TILs	Indicates ongoing immune response in tumors.	High TILs improve response to ICIs.	Predictive of better survival in metastatic TNBC.
LAG3	Inhibitory receptor on T and NK cells.	Enhanced response when combined with PD-1 blockade.	Potential target in combination therapies.
TIM3	Inhibits various immune cells.	Regulates immune responses.	Explored as a co-target with other checkpoints.
TMB	Quantifies mutation burden in tumors.	High TMB linked to ICI sensitivity.	Limited value in TNBC; high TMB in 3% of primary and 11% of metastatic cases.
MSI	Results from DNA replication errors.	Rare in TNBC.	Limited clinical utility in TNBC.
Cytokines (e.g., IL-8, VEGF)	Modulate tumor growth and immune response.	High VEGF and IL-8 promote tumor progression.	Serve as prognostic markers; potential therapeutic targets.
Blood Cytokine Profile	Includes IL-1α, TRAIL, SCF, CCL5, IL-16.	Levels correlate with treatment outcomes.	Predictive of better outcomes when high during treatment.

MSI, microsatellite instability; TILs, tumor-infiltrating lymphocytes; TMB, tumor mutational burden.

### 3.3 Advanced imaging technologies

In parallel with molecular and cellular biomarkers, advanced imaging technologies are increasingly employed to spatially map immune responses within tumors. Multispectral imaging platforms, such as AstroPath, combine traditional histology with quantitative measurement of multiple immune and tumor markers *in situ*. This spatial resolution allows for the identification of key microanatomical features—such as the presence of immune cell niches, tumor-immune boundaries, or exclusion zones—that may influence immunotherapy efficacy. Such insights are critical for interpreting treatment-induced remodeling of the tumor microenvironment in TNBC (166).

#### 3.3.1 Immuno-positron emission tomography

Immuno-positron emission tomography (immuno-PET) represents a complementary strategy to evaluate immune responses non-invasively. By radiolabeling therapeutic antibodies such as pembrolizumab (^89^Zr-pembrolizumab), researchers can visualize *in vivo* binding of the antibody to PD-1-expressing cells within tumors or lymphoid tissues. Early studies in NSCLC and melanoma have demonstrated the feasibility of this approach, and its translation to BC is currently under investigation (167).

#### 3.3.2 Radiomics and delta radiomics

Radiomics and delta radiomics further expand the landscape of imaging-based biomarkers by analyzing subtle changes in texture, shape, and intensity within CT or PET scans using artificial intelligence tools. These features, invisible to the human eye, can reflect underlying biological processes such as immune infiltration, fibrosis, or necrosis. Machine learning algorithms trained on radiomic datasets have shown promise in predicting treatment response in various cancers (168). In the context of TNBC, delta radiomics could provide an early, non-invasive readout of immunological changes induced by neoadjuvant therapies, potentially guiding therapy adjustments.

#### 3.3.3 Advanced spatial tissue profiling

Complementing systemic imaging, advanced spatial profiling techniques allow high-resolution characterization of the tumor immune microenvironment directly within tissue architecture. Multiplex immunofluorescence (mIF) enables simultaneous detection of multiple proteins on the same tissue section, providing quantitative insights into the spatial organization and interactions of immune cells. Spatial transcriptomics approaches, such as 10x Genomics Visium or NanoString GeoMx, map gene expression across defined tissue coordinates, capturing localized immune signatures and stromal-immune crosstalk. Highly multiplexed imaging platforms, including CODEX and digital pathology workflows, allow comprehensive mapping of dozens of markers while preserving tissue context. Together, these technologies reveal functional niches, immune exclusion zones, and cellular neighborhoods that cannot be resolved by conventional IHC or bulk molecular assays. Integration of spatial profiling with immuno-PET and radiomics offers a multidimensional view, linking systemic imaging with tissue-level cellular and molecular architecture, ultimately enhancing the precision of immune monitoring and patient stratification in TNBC immunotherapy trials.

### 3.4 Temporal biomarker sampling

The temporal aspect of immune monitoring is equally crucial. Immune responses evolve dynamically over the course of treatment, necessitating longitudinal sampling strategies. In responders to neoadjuvant chemo-immunotherapy, early immunological events often include a surge in effector T-cell activity, increased IFN-γ signaling, and reprogramming of macrophages toward a proinflammatory phenotype (167). These changes are typically accompanied by reductions in tumor burden and the development of immunological memory. In contrast, non-responders may exhibit persistent immunosuppressive features, such as sustained presence of regulatory myeloid populations, impaired antigen presentation, or T-cell exclusion from the tumor core. Serial monitoring through tissue biopsies, blood sampling, or imaging enables the detection of these divergent trajectories, informing timely therapeutic modifications (169, 170).

### 3.5 Patient-derived models

To support real-time functional precision medicine, novel platforms such as zebrafish avatars are being developed. In this model, patient-derived tumor cells are engrafted into immunocompromised zebrafish embryos, which then serve as rapid screening platforms for therapeutic efficacy. Initial studies in colorectal cancer have demonstrated high concordance between zebrafish responses and patient outcomes to chemotherapy (171). Extending this approach to TNBC may offer insights into potential treatment responses, including immunotherapy or combinatorial regimens, but clinical validation remains limited. Key challenges include scalability for larger patient cohorts, standardization of engraftment and readout protocols, and ensuring reproducibility across laboratories.

Similarly, the TRIPLEX study (172) is investigating the feasibility of generating patient-derived tumor organoids (PDTOs) from TNBC biopsies to assess their ability to predict clinical response to chemotherapy and immune checkpoint blockade. While these models provide a promising ex vivo platform to interrogate tumor biology, several limitations remain: the generation and maintenance of PDTOs can be labor-intensive, time-consuming, and technically demanding; variability between organoids derived from different patients may affect generalizability; and integration of PDTO-derived data into clinical decision-making pathways is not yet established. Consequently, these platforms are currently better suited for exploratory studies and mechanistic investigations rather than routine clinical use.

In conclusion, while zebrafish avatars and PDTOs represent innovative tools for functional precision oncology, their current clinical utility in TNBC is still largely investigational. Integration of these models with tissue-based, blood-derived, imaging-driven, and temporally resolved biomarkers provides a multidimensional perspective of the immune response to therapy (**Table 3**). Future efforts should focus on rigorous validation, optimization for scalability, and establishing standardized pipelines to facilitate translation into personalized treatment strategies. Complementary incorporation of advanced multiplex technologies, artificial intelligence, and integrative modeling will be essential to refine predictive frameworks and improve patient outcomes in this aggressive BC subtype.

**Table 3:** Biomarker-based immune monitoring strategies in cancer immunotherapy.

Biomarker type	Specific biomarker	Function / Use	Application in TNBC	Key references
Tissue-based	PD-L1 (TPS/CPS)	Immune checkpoint ligand; predictive of ICB response	Approved for use in selecting patients for anti–PD-1/PD-L1 therapy	(98, 104)
	MSI/dMMR	Reflects genomic instability and neoantigen load	Rare in TNBC; predicts strong ICB response when present	(113, 116)
	Tumor Mutational Burden (TMB)	Measures somatic mutations per Mb of DNA; proxy for neoantigen load	High TMB may correlate with ICB response in select patients	(118, 123)
	IFN-γ Gene Signatures	Measures IFN-γ pathway activation; immunogenicity marker	Potential to stratify responders to neoadjuvant immunotherapy	(105, 143)
Blood-based	Cytokines & Soluble Proteins	Track systemic immune activation / toxicity	Dynamic, longitudinal monitoring of therapy effects	(145)
	Immune-related Gene Expression	Transcriptomic analysis of PBMCs / CTCs	Monitors immune activation, exhaustion, or suppression	(105)
	Comprehensive Immunophenotyping	Deep immune profiling (CyTOF, flow cytometry)	Tracks immune reconstitution and correlates with response/non-response	(147)
Imaging-based	Multispectral Imaging (AstroPath)	Spatial resolution of immune-tumor architecture	Identifies immune niches, exclusion zones	(166)
	ImmunoPET	*In vivo* tracking of PD-1/PD-L1 expression	Non-invasive biomarker of immune dynamics	(173)
	Radiomics / Delta Radiomics	AI-driven analysis of CT/PET image features	Early prediction of immunologic changes and treatment response	(168)
Temporal profiling	Serial biopsies & blood sampling	Captures dynamic immune shifts during treatment	Differentiates responders vs. non-responders; supports adaptive therapy	(167, 170)
Functional models	Zebrafish avatars	Rapid *in vivo* drug screening using patient-derived xenografts	Forecasts individual responses; emerging in TNBC	(171)

CPS, Combined Positive Score; CTC, circulating tumor cells; CyTOF, Cytometry by Time-of-Flight; ICB, immune checkpoint blockade; ImmunoPET, Immuno-positron emission tomography; MSI , microsatellite instability; MMR, DNA mismatch repair; PBMCs; peripheral blood mononuclear cells; TPS, Tumor Proportion Score.

## 4 Future directions and emerging techniques

Immune monitoring involves evaluating the function, activity, and state of the immune system by analyzing immune cells, cytokines, and biomarkers. This is crucial for understanding disease progression, response to therapies, and predicting outcomes. Traditional immune monitoring methods, such as flow cytometry and ELISA, have been foundational. However, recent innovations have significantly expanded the capability to assess immune responses with greater precision and detail.

### 4.1 Single-cell sequencing

Single-cell sequencing is a next-generation sequencing (NGS) method that examines the genomes or transcriptomes of individual cells, providing a high-resolution view of cell-to-cell variation (176). In cancer, the process of sequencing the DNA of individual cells can provide valuable insights about the mutations that are present in isolated cell groups. The sequencing of RNAs produced by individual cells throughout development can provide valuable understanding of the presence and characteristics of various cell types (177).

Profiles of gene expression in bulk tumors reveal the characteristics of non-tumor compartments, which, in the case of BC, are marked by a significant combination of stromal, immunological, and endothelial cell infiltration. The admixtures constitute the tumor microenvironment and have a crucial function in the initiation, development, and resistance to therapy of tumors. Prognostic values of micro-environmental gene expression profiles may exist independently of the underlying tumor subtype (178–180). The primary cellular components of the cancer microenvironment consist of cancer-associated fibroblasts and immune cells. The cancer microenvironment comprises cancer-associated fibroblasts and diverse complement of immune cells. In general, tumor-associated macrophages (TAMs) facilitate the proliferation and spread of tumors, whereas CD8+ cytotoxic T cells and CD4+ Th1 cells provide support for the immune response against tumors (181). There is a correlation between regulatory or exhausted T cells and unsuccessful anticancer responses. Despite the potential contribution of certain B cells to the advancement of tumors, a significant abundance of B cells within the tumor area is linked to a positive prognosis (182, 183). Fundamentally, the tumor microenvironment is influenced by the interactions between these heterogeneous cells and cancer cells.

Single-cell genome analysis is anticipated to play a significant role in cancer treatment. It can aid in non-invasive monitoring of circulating tumor cells, assessing tumor heterogeneity, detecting small recurrent tumors early, and monitoring rare cell populations in difficult-to-treat cancers. Understanding transcriptome heterogeneity and accurately characterizing gene expression in tumors and their microenvironments may help identify more effective molecular targets for prognosis and treatment (184). Characterizing tumor heterogeneity could lead to targeted therapies, while profiling tumor-infiltrating immune cells may offer new strategies to combat immune suppression and enhance immune surveillance (185).

TNBC exhibits significant intra- and inter-tumor heterogeneity, complicating treatment and prognosis. Single-cell sequencing (SCS) has been instrumental in identifying distinct TNBC cell types, such as basal and other epithelial cells, which are linked to poor survival outcomes. SCS also reveals genetic mutations and clonal evolution within TNBC, helping to understand tumor resistance during chemotherapy and uncover new treatment targets. Moreover, SCS has exposed the heterogeneity of cancer-associated fibroblasts (CAFs) and immune cells, crucial in tumor progression and drug resistance. This technique also supports the exploration of combination therapies and personalized approaches, offering potential strategies to reduce recurrence, metastasis, and treatment resistance in TNBC (186).

### 4.2 Circulating tumor DNA and liquid biopsy

Liquid biopsy is a minimally invasive technique that has gained significant attention in recent years for its utility in cancer diagnostics, prognosis, and treatment monitoring. It involves the analysis of peripheral blood or other bodily fluids to detect tumor-derived components such as circulating tumor cells (CTCs), cell-free DNA (cfDNA), and circulating tumor DNA (ctDNA). Notably, ctDNA represents a tumor-derived fraction of the total cfDNA. Unlike traditional tissue biopsy, liquid biopsy can be performed repeatedly with minimal discomfort, making it a valuable tool for real-time and longitudinal assessment of tumor dynamics (187). ctDNA, a form of circulating nucleic acid derived from cancer cells, is typically released through apoptosis, necrosis, or active secretion. Apoptosis is the most common mechanism, producing nucleosome-protected DNA fragments approximately 140–180 base pairs in length (188, 189). Additionally, nucleic acids may circulate as free fragments or encapsulated within extracellular vesicles, offering a window into the molecular characteristics of both primary and metastatic tumors.

In the broader oncology landscape, ctDNA and CTCs—collectively referred to as the “circulome”—are increasingly recognized as biomarkers for disease progression, treatment resistance, and measurable residual disease (MRD). High levels of ctDNA have been correlated with aggressive disease phenotypes and relapse across multiple cancer types. Moreover, circulating DNA fragments have been associated with tumor size and lymph node involvement, indicating their utility for staging and risk stratification (190, 191). In advanced cancers, liquid biopsy enables molecular characterization of therapy-resistant clones, helping clinicians tailor treatments in a personalized manner (192, 193). As such, liquid biopsies serve as a complementary tool to traditional tissue biopsies, offering additional, dynamic insights into the tumor’s genetic evolution.

In TNBC, where tumor heterogeneity and lack of targeted therapies pose significant challenges, ctDNA and liquid biopsy offer promising avenues for disease monitoring and precision oncology. An increase in ctDNA levels has been linked to aggressive subtypes and poorer prognoses. One large study involving 130 TNBC patients measured ctDNA levels within seven months post-treatment and found that 7.7% had detectable ctDNA, signifying residual disease. Notably, among patients who had undergone neoadjuvant therapy but did not achieve pCR, the presence of ctDNA was associated with shorter PFS, highlighting the prognostic value of ctDNA in identifying high-risk patients (194). These findings underscore the benefit of integrating ctDNA monitoring with tissue-based response to refine post-treatment surveillance and decision-making in TNBC.

Further, ctDNA has shown promise as a dynamic biomarker during neoadjuvant therapy. Riva et al. investigated the use of TP53 mutation-based digital PCR assays to monitor ctDNA in early-stage TNBC patients (195). ctDNA was detectable in 75% of patients at baseline, with levels decreasing throughout treatment, except in one patient who exhibited disease progression. Interestingly, although ctDNA levels did not correlate directly with clinical response or pCR, the absence of ctDNA post-surgery suggested favorable outcomes. The limited sensitivity in this study was likely due to tracking a single mutation. In contrast, a follow-up study by Cavallone et al. enhanced ctDNA detection by targeting an average of five mutations per patient (196). In this cohort, ctDNA was detectable in 96% of patients at baseline, and pre-surgical ctDNA positivity was associated with residual disease and inferior long-term outcomes. Conversely, patients with undetectable ctDNA before surgery had improved relapse-free and overall survival, reinforcing the predictive and prognostic value of multiplexed ctDNA monitoring in TNBC (196).

Importantly, clinical trials have now applied ctDNA monitoring to guide early detection of recurrence and treatment adaptation. The c-TRAK TN trial (174) demonstrated that serial ctDNA assessment post-neoadjuvant therapy could identify MRD prior to clinical relapse, with ctDNA-positive patients showing a significantly higher risk of recurrence. Similarly, the ZEST trial (175) confirmed that ctDNA dynamics could guide adjuvant treatment decisions, with early clearance associated with improved relapse-free survival. These studies highlight the growing utility of ctDNA as a predictive and prognostic biomarker and support its integration into personalized surveillance strategies in TNBC immunotherapy trials (**Table 4**).

**Table 4:** Overview of landmark neoadjuvant chemoimmunotherapy trials in TNBC: trial design, biomarker integration, and outcome measures.

Trial	Phase	ICI / agent & schedule	PD-L1 assay / cutoff	TILs	ctDNA results	Primary outcomes (pCR/EFS/OS)	Reference
KEYNOTE-522	III	Pembrolizumab with neoadjuvant chemo and adjuvant continuation	22C3 CPS (exploratory); benefit irrespective of CPS	Higher TILs associated with higher pCR	Exploratory analyses ongoing	pCR 64.8% vs 51.2%; 36-mo EFS HR 0.63; 60-mo OS 86.6% vs 81.7%	(71, 84, 36, 36)
IMpassion031	III	Atezolizumab with neoadjuvant chemo (perioperative)	SP142; ≥1% immune cells considered positive	TIL-rich tumors showed greater benefit trends	Not primary endpoint; exploratory analyses reported	pCR 58% vs 41%; no mature EFS benefit	(99)
NeoTRIP Michelangelo	III	Atezolizumab with carboplatin + nab-paclitaxel (pre-AC)	Assays varied; exploratory analyses	Biomarker context affected response; TILs informative	Exploratory	pCR 48.6% vs 44.4% (NS)	(100)
GeparNuevo	II	Durvalumab (window then with chemo)	PD-L1 and TILs analyzed; serial biopsies	Higher baseline TILs correlated with response; dynamic changes informative	Subset analyses	Modest pCR increase; follow-up showed improved iDFS/DDFS in some analyses	(101)
BrighTNess	III	Veliparib + carboplatin vs carboplatin alone vs control (no veliparib)	N/A (not ICI primary)	BRCA/HRD tumors had higher benefit from carboplatin	Exploratory	Carboplatin improved pCR and EFS; veliparib did not add benefit	(95)
KEYNOTE-173	Ib	Pembrolizumab with various neoadjuvant regimens	22C3 CPS and TILs assessed	Higher TILs and PD-L1 correlated with higher pCR in exploratory cohorts	Exploratory correlative work	Promising pCR rates in several cohorts; informed KEYNOTE-522 design	(71)
IMpassion130	III	Atezolizumab + nab-paclitaxel (first-line metastatic)	SP142 on immune cells; PD-L1+ defined as IC≥1%	PD-L1+ / TIL-rich tumors derived more PFS benefit	Exploratory analyses	PFS benefit in PD-L1+ subgroup; OS benefit subject to follow-up and regulatory considerations	(111)
NeoSTAR (Arm A2)	II	Sacituzumab govitecan (10 mg/kg d1,8) + pembrolizumab (200 mg d1) ×4 cycles; ANACT allowed	Exploratory; BRCA subgroup reported	Activity observed across biomarker subgroups; higher in some immune-active tumors	Not primary endpoint; biomarker work planned	pCR after SG+P alone 34%; overall pCR 50% after ANACT; 60% pCR in BRCA-mutant subset (3/5)	(102)
c-TRAK TN	II	Intervention triggered by ctDNA detection (varies)	Not directly applicable	NA	Serial ctDNA detected MRD prior to clinical relapse; ctDNA+ associated with higher recurrence risk	Study focused on MRD detection and feasibility of intervention; ctDNA preceded relapse in many cases	(174)
ZEST	III	Niraparib vs placebo guided by ctDNA status (adjuvant)	NA	NA	Design uses ctDNA to guide adjuvant therapy; early reports indicate ctDNA dynamics associate with outcomes	Ongoing; presented as abstract at SABCS 2024	(175)

AC, Anthracycline-cyclophosphamide; ANACT, Anthracycline-containing neoadjuvant chemotherapy; BRCA , Breast cancer gene (BRCA1/2); ctDNA, Circulating tumor DNA; DDFS, Distant disease-free survival; EFS, Event-free survival; HER2-, Human epidermal growth factor receptor 2 negative; iDFS, Invasive disease-free survival; ICI, Immune checkpoint inhibitor; MRD, Minimal residual disease; NA, not applicable; NACT, Neoadjuvant chemotherapy; nab paclitaxel , Nanoparticle albumin-bound paclitaxel; OS, Overall survival; PD-L1+, Programmed death-ligand 1 positive; pCR0, Pathologic complete response; PFS, Progression-free survival; SG, Sacituzumab govitecan; TNBC, Triple-negative breast cancer; d1, d8, Day 1, Day 8 of treatment cycle; I, Ib, II, III ,Clinical trial phases.

## 5 Limitations and controversies of key tissue biomarkers and clinical implications for TNBC

Despite their extensive investigation, PD-L1, TMB, and MSI each present important limitations that diminish their ability to function as reliable stand-alone predictive biomarkers in TNBC.

PD-L1 illustrates both technical and biological heterogeneity. Different immunohistochemical assays (such as 22C3, SP142, and SP263) employ non-equivalent scoring systems—tumor proportion score, combined positive score, or immune cell scoring—leading to discordant classifications of the same tumor. A specimen considered “positive” by one assay may be deemed “negative” by another (69, 100). Beyond these analytical inconsistencies, PD-L1 expression is highly variable within the same tumor, between primary and metastatic lesions, and is subject to dynamic regulation by therapy and inflammatory cues. As a result, reliance on a single archival biopsy may not accurately capture the current biological state of the disease (106). This variability translates into significant clinical consequences. In metastatic TNBC, PD-L1 status is routinely used to guide the use of ICIs, as in KEYNOTE-355 where pembrolizumab was restricted to CPS-positive disease. In contrast, in the neoadjuvant setting, KEYNOTE-522 demonstrated benefit from pembrolizumab regardless of PD-L1 expression, creating uncertainty about the biomarker’s role in treatment selection at earlier disease stages (71, 84). In practice, such inconsistencies risk both undertreatment, by denying effective therapy to some patients, and overtreatment, by exposing others to toxicity without clear evidence of benefit.

TMB faces a different set of challenges. Measurement varies depending on sequencing approach, bioinformatics pipeline, and the genomic territory interrogated, whether whole exome or targeted panels. These methodological inconsistencies lead to divergent mutational burden estimates and complicate the establishment of universal thresholds (118). Although the FDA’s pan-tumor approval of pembrolizumab for TMB ≥10 mut/Mb, based on KEYNOTE-158, provided a proof-of-concept for its utility, subsequent data revealed limited reproducibility across tumor types and very little evidence specific to TNBC (123). In breast cancer overall, high TMB is rare, and when present, does not consistently predict durable responses to checkpoint blockade (120, 124). Consequently, TMB alone provides limited clinical confidence. Its potential value lies in integration with other features of the immune landscape, such as tumor-infiltrating lymphocytes, interferon-gamma–related gene expression signatures, and clonal architecture of the tumor.

MSI or mismatch repair deficiency is another theoretically appealing biomarker because MSI-high tumors are strongly immunogenic and respond well to PD-1 blockade across multiple cancers (113, 114). However, MSI-H status is exceedingly rare in breast cancer, and particularly in TNBC, with a prevalence below 2% (115, 116). This rarity sharply limits its clinical utility as a broad biomarker in this disease. Moreover, MSI detection itself is not straightforward, discordance can occur between testing methods, including immunohistochemistry for mismatch repair proteins, PCR–based microsatellite panels, and NGS–derived MSI calls. In a low–prevalence context, even modest rates of false positives or false negatives carry disproportionate clinical consequences. While MSI positivity should always prompt consideration of PD-1 therapy regardless of tumor origin, routine MSI screening in all TNBC cases has limited yield.

Taken together, these three biomarkers share fundamental limitations. Pre-analytic factors such as tissue handling, fixation, and ischemia time can influence results, while intratumoral heterogeneity and sample type further complicate interpretation. A single static biomarker measurement fails to reflect the evolving interaction between the tumor and immune system during therapy. Lack of assay harmonization across platforms prevents direct comparability between studies and undermines reproducibility. These weaknesses have practical clinical implications: in metastatic disease, reliance on PD-L1 to determine eligibility for immunotherapy may exclude some patients who could benefit, while in early TNBC the proven efficacy of immunotherapy regardless of PD-L1 status makes such selection criteria questionable. Similar risks arise with TMB and MSI, where methodological or prevalence-related limitations may lead to inappropriate treatment denial or unwarranted exposure to toxicity.

Addressing these challenges requires pragmatic strategies. The use of drug-specific, validated assays and cutoffs is essential for clinical decision-making. Testing should be performed on the most relevant sample, ideally the most recent and representative of the disease stage being treated. Overreliance on any single biomarker should be avoided in favor of integrative approaches that combine PD-L1 status with tumor-infiltrating lymphocytes, immune-related gene signatures, and liquid biopsy markers such as circulating tumor DNA. Longitudinal and repeated sampling in the trial setting may help to capture dynamic biomarker changes, while ongoing efforts in assay harmonization and cross-platform benchmarking will be critical to improving comparability between studies. Ultimately, progress in TNBC immunotherapy will depend on the development of composite, multimodal biomarker frameworks that integrate spatial, transcriptomic, and circulating immune readouts, offering a more accurate reflection of tumor–immune interactions and greater predictive precision than any single analyte can provide.

## 6 Conclusion

Immune monitoring has become an important strategy that can further elucidate neoadjuvant chemo-immunotherapy responses in TNBC, a tumor type with limited therapeutic options and generally characterized by a poor prognosis. By evaluating immune markers such as TILs, cytokine profiles, and immune checkpoint expression, clinicians gain real-time insights into how the immune system interacts with the tumor, guiding adjustments to treatment strategies and improving outcomes. For example, higher levels of TILs have been associated with better responses, therefore underlining the importance of a strong immune presence in enhancing the efficacy of chemo-immunotherapy.

The combination of chemotherapy and immunotherapy holds significant promise due to their complementary mechanisms of action. Chemotherapy induces tumor cell death, releasing antigens that can stimulate the immune system, while immunotherapy enhances the immune response by preventing immune evasion. Immune monitoring can track the effectiveness of this synergy, ensuring that both arms of therapy are functioning optimally.

Despite its potential, immune monitoring in TNBC faces several challenges. Tumors can evolve rapidly, and immune markers may fluctuate, complicating long-term predictions. The pronounced biological heterogeneity of TNBC—encompassing variations in tumor-intrinsic features, immune microenvironments, and patient-specific factors—makes it particularly difficult to develop universal biomarkers that reliably predict responses across all patients. In addition, practical limitations—including high costs of advanced assays, variability in assay reproducibility, and limited access to high-throughput technologies and specialized expertise—pose barriers to routine clinical implementation.

Emerging technologies like next-generation sequencing and single-cell analysis are beginning to unravel this complexity, paving the way for more personalized and accurate immune monitoring. While there are still hurdles to overcome, ongoing research and technological advancements are likely to refine immune monitoring techniques, enhancing their predictive power and clinical utility. As our understanding of the immune system’s role in cancer therapy deepens, immune monitoring is expected to play an increasingly central role in the management of TNBC and other cancers, ultimately leading to better patient care and improved survival outcomes.
